# Aseptic leptomeningitis induced by azathioprine in systemic lupus erythematosus: a rare manifestation

**DOI:** 10.1259/bjrcr.20230063

**Published:** 2023-09-11

**Authors:** Mariana Santos, Matheus Alves da Silva, Maria Laura Piassa, Danielly Santos, Alex Machado Baeta, Lázaro Luis Amaral

**Affiliations:** 1 Neuroradiology Department, Hospital de Braga, Braga, Portugal; 2 Neurology Department, Beneficiência Portuguesa de São Paulo, São Paulo, Brazil; 3 Radiology Department, Beneficiência Portuguesa de São Paulo, São Paulo, Brazil

## Abstract

Systemic lupus erythematosus (SLE) is an autoimmune systemic disease and these patients can have neurological involvement; however, aseptic leptomeningitis is considered to be a very rare feature, observed in 1.4–2.0% of patients. Here, we described a case of a young male with SLE treated with azathioprine with progressive headache, which revealed diffuse posterior fossa leptomeningitis, relatively sparing the supratentorial compartment, that represent an adverse drug reaction – a rare manifestation of central nervous system involvement in SLE. Treatment with azathioprine was interrupted and methylprednisolone was initiated and the patient has significant improvement of his neurological state in 5 days later, demonstrating total involution of the leptomeningeal enhancement on MRI follow-up.

## Clinical presentation

A 25-year-old male presented to the emergency department with a 2-week history of refractory headache. He described a periorbital pulsatile headache with progressive worsening, associated with nausea, vomiting and photophobia. The patient denied other relevant symptoms. Neurological examination was unremarkable and showed no signs of meningism. His medical history was positive for systemic lupus erythematosus (SLE), discovered after investigation of deep vein thrombosis associated with polyarthritis and skin lesions. He started taking azathioprine a few months before the headache symptoms onset. Neurological examination was unremarkable without motor deficits or signs of meningeal inflammation.

## Investigations

**Figure 1.**
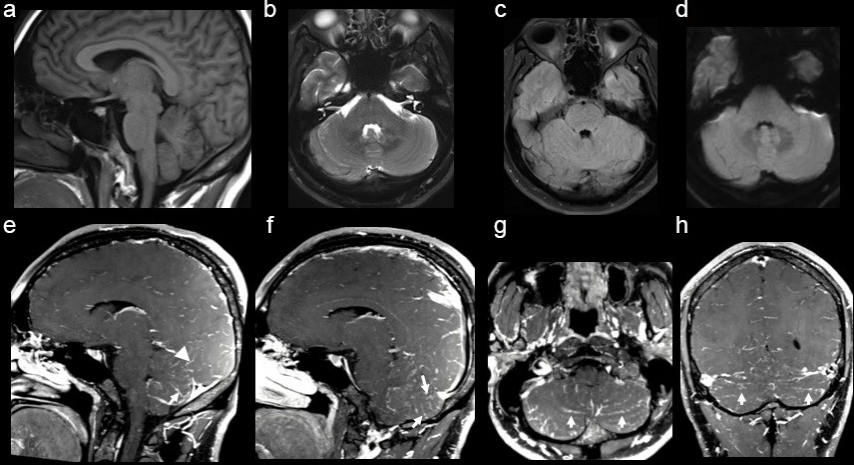
(a) Sagittal MRI *T*_1_-weighted images, (b) axial T2-weighted images, (c) axial T2-FLAIR MRI and axial diffusion-weighted images (DWI), (d) show no abnormal findings. Sagittal (**e and **f), axial, (g) and coronal, (h) T1-weighted images post-contrast depict diffuse and bilateral leptomeningeal enhancement in posterior fossa between cerebellar *folia* (*arrows*) and in occipital lobes, less extensively (*arrowhead,* e).

Non-contrast-enhanced brain computed tomography (CT) exam showed no relevant alterations. A brain magnetic resonance imaging (MRI) (Figure 1) was performed for further evaluation, demonstrating bilateral diffuse enhancement along the cerebellar folia on *T*_1_-weighted post-contrast imaging and, to a lesser extent, in occipital lobes, without restricted diffusion or hyperintense signal on T2/FLAIR images. These findings were suggestive of leptomeningitis. Lumbar puncture was performed, revealing slight increase in opening pressure (24 cm H_2_O) and in protein content in cerebral spinal fluid (CSF), 89 mg dl^−1^, with increased number of cells (181 cells/cm^3^, with predominance of lymphocytes) and normal levels of glucose. CSF analysis were negative for bacterial microorganisms, tuberculosis, virus and neoplastic process.

## Differential diagnosis

The main differential diagnosis for leptomeningitis is an inflammatory/infectious central nervous system process or carcinomatous (secondary to a primary malignancy). In this particular case, the clinical status of the patient and the innocent laboratory study, most likely, suggested an inflammatory process related to the patient’s underlying disease – SLE.

## Treatment

After the MRI, we started empiric acyclovir and ceftriaxone, thinking about infectious aetiology. After CSF analyses were negative for bacterial microorganisms, tuberculosis, virus and neoplastic cells and, after the panel was negative for infectious aetiologies, these drugs were stopped. His SLE showed no signs of systemic activity with no skin lesions, arthralgia, visceral involvement or haematological abnormalities, with normal inflammatory markers, like c reactive protein and erythrocyte sediment rate, so we suspected aseptic meningitis due to a pharmacological aetiology. Treatment with azathioprine was interrupted and methylprednisolone was initiated at a dosage of 60 mg/day.

## Outcome and follow-up

**Figure 2.**
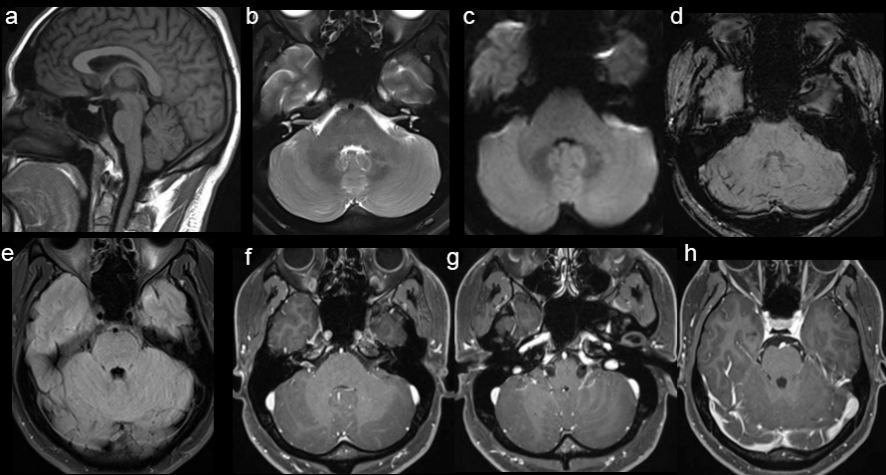
(a) Sagittal MR T1-weighted images, (b) axial T2-weighted images, (c) axial DWI, (d) axial susceptibility-weighted images (SWI) and (e) axial T2-FLAIR MRI continued demonstrating no abnormalities. (**f, g and **h) Axial *T*_1_-weighted images post-contrast show complete resolution of the leptomeningeal enhancement 5 days after drug withdrawal and corticosteroid therapy.

The patient has significant improvement of his neurological state. His second MRI demonstrated total involution of the leptomeningeal enhancement five days after the drug was withdrawn (Figure 2).

## Discussion

SLE is a chronic autoimmune inflammatory condition, most frequently seen in young women.^1^ Although the literature reports central nervous system involvement in patients with SLE, aseptic leptomeningitis is considered to be a very rare feature, observed in 1.4–2.0% of patients,^2^ presenting with leptomeningeal enhancement on brain MRI post-contrast images but without poor clinical status or positive infectious (bacterial, tuberculous or viral) CSF cultures.^3,4^ The presumed cause of this aseptic (non-infectious) leptomeningitis is thought to be a dysregulation in clearance the immune complexes, with increased inflammatory molecules, subsequently leading to infiltrate inflammatory in tissues, according to autoimmune patophysiology.^1,5^ Nonsteroidal anti-inflammatory drugs and immunosuppressive agents are commonly used in the treatment of patients with SLE and can be at the origin of these central nervous system findings on MRI.^6^ To our knowledge, there are few cases described in the literature reported aseptic leptomeningitis induced by azathioprine.^7^

So, in our opinion, brain MRI plays an important role in the diagnosis of this entity, which has a characteristic leptomeningeal enhancement on *T*_1_-weighted images, without signs of infectious disease such as hydrocephalus secondary to a bacterial meningitis or parenchymal lesions related to secondary vasculitis in a patient who is mildly symptomatic. CSF analysis and exclusion of other possible causes, such as leptomeningeal carcinomatosis, are other important features in the diagnosis of this entity.^8^

We believe that our case represents an adverse drug reaction, with an unusual manifestation of central nervous system in patients with SLE, which makes it even more important to perform brain MRI in a patient with this autoimmune multisystemic disease.

## Learning points

- This case emphasises that complex autoimmune diseases have the potential for rare complications.
- Leptomeningitis in patients with SLE can be secondary to a dysregulation in clearance of the immune complexes, according to an inherent autoimmune pathophysiology or related to drugs.
- Brain MRI is an invaluable diagnostic in the workup of azathioprine-related aseptic leptomeningeal enhancement and ruling out infectious causes.

## References

[1] Tsokos GC . Systemic lupus erythematosus. N Engl J Med 2011; 365: 2110–21. doi: 10.1056/NEJMra1100359 22129255

[2] Tsukamoto M, Shimamoto M, Terashima T, Seta M . Aseptic meningitis with systemic lupus erythematosus: case report and review of the literature. Arch Rheumatol 2019; 34: 108–11. doi: 10.5606/ArchRheumatol.2019.7026

[3] Lee JH, Lee JY, Lee YJ, Park DW, Kim YS, Kim HY . Noninfectious meningitis caused by systemic lupus erythematosus. J Comput Assist Tomogr 2016; 40: 424–27. doi: 10.1097/RCT.0000000000000386 26938698

[4] Roccatello D, Emmi L . Connective tissue disease: a comprehensive guide. Springer 2016; 1. doi: 10.1007/978-3-319-24535-5

[5] Schwartz N, Stock AD, Putterman C . Neuropsychiatric lupus: new mechanistic insights and future treatment directions. Nat Rev Rheumatol 2019; 15: 137–52. doi: 10.1038/s41584-018-0156-8 30659245 PMC8023338

[6] Brey RL, Holliday SL, Saklad AR, Navarrete MG, Hermosillo-Romo D, Stallworth CL, et al . Neuropsychiatric syndromes in lupus: prevalence using standardized definitions. Neurology 2002; 58: 1214–20. doi: 10.1212/wnl.58.8.1214 11971089

[7] Sergent JS, Lockshin M . Azathioprine-induced meningitis in systemic lupus erythematosus. JAMA 1978; 240: 529. doi: 10.1001/jama.240.6.529c 671659

[8] Hanly JG, McCurdy G, Fougere L, Douglas J-A, Thompson K . Neuropsychiatric events in systemic lupus erythematosus: Attribution and clinical significance. J Rheumatol 2004; 31: 2156–2162. 15517627

